# Iliopsoas Abscess Mimicking a Lower Motor Neuron Lesion: A Diagnostic Challenge

**DOI:** 10.7759/cureus.73978

**Published:** 2024-11-19

**Authors:** Pallavi Priya, Aravind Nair, Sathyanarayana Gowda, Shahid Nasim, Vinod Warrier

**Affiliations:** 1 Internal Medicine, Mid and South Essex NHS Foundation Trust, Southend-on-Sea, GBR; 2 Acute Medicine, Mid and South Essex NHS Foundation Trust, Southend-on-Sea, GBR

**Keywords:** diabetic ketoacidosis (dka), diabetic uropathy, iliopsoas abscesses, immunocompromised individuals, ultrasound-guided abscess drainage

## Abstract

Iliopsoas abscess is a rare infection that presents with a triad of fever, back pain, and hip pain. However, due to the anatomical proximity of the psoas muscle to various structures, an abscess in this region can manifest with nonspecific symptoms, leading to potential misdiagnosis and delayed diagnosis, which can be fatal. We report a case of a 54-year-old female who presented to the emergency department with right-sided flank pain and symptoms resembling lower motor neuron disorder. Initial investigations were inconclusive until an MRI revealed a large retroperitoneal collection in the right perinephric space and iliopsoas muscle, likely of urological origin, with no spinal abnormalities. Intravenous gentamicin was initiated. A subsequent contrast-enhanced CT scan of the abdomen and pelvis confirmed a right posterior perinephric collection. Ultrasound-guided drainage was performed, and a pigtail catheter was placed in situ for continuous drainage along with targeted antibiotics based on culture and sensitivity.

## Introduction

Iliopsoas abscess (IPA) is an uncommon condition with a diverse range of clinical presentations, often making diagnosis challenging. Due to the anatomical proximity of the psoas muscle to various structures, an abscess in this region can manifest with nonspecific symptoms, leading to potential misdiagnosis [1]. The incidence of IPA in the United Kingdom is approximately 0.4 cases per 100,000 people. However, atypical presentations complicate diagnosis and may contribute to underreporting [1]. Only about 30% of cases present with the classic triad of symptoms: fever, back pain, and hip pain, preventing early diagnosis and leading to potential delays in treatment [2]. This case report highlights a 54-year-old woman who presented with symptoms suggestive of a lower motor neuron (LMN) lesion, which was later identified as IPA.

## Case presentation

We present the case of a 54-year-old female who presented to the emergency department with complaints of right flank pain radiating to the thigh, accompanied by lower back pain of three weeks duration. She reported intermittent numbness and paraesthesia in her right buttock and right thigh, as well as a significant reduction in bowel movements during this period. Her symptoms also included decreased mobility, reduced appetite, and diminished fluid intake.

She has a background of type 2 diabetes mellitus, hypertension, long QT syndrome, recurrent abscesses, diabetic retinopathy, overactive bladder, and depression. The patient lives in a shared accommodation and is known to be noncompliant with her insulin.

Upon arrival at the hospital, the patient's vital signs revealed a temperature of 37.1℃, a heart rate of 87 beats/minute, a blood pressure of 104/64 mmHg, and a SpO₂ of 98% on room air. A cardiovascular examination revealed regular heart sounds with no murmurs, and respiratory sounds were clear with no adventitious sounds.

On neurological examination, the patient was observed to have a limping gait and reduced muscle strength in right hip flexion and external rotation. Palpation revealed tenderness over the right flank, and there was a marked reduction in anal sphincter tone, though perianal and lower limb sensations remained equal and intact in both lower limbs. Reflexes were not assessed initially as the patient declined a full neurological examination due to significant pain.

On admission, venous blood gas showed a pH of 7.21, glucose 27.8 mmol/L, ketones 3.6 mmol/L, and lactate 0.9 mmol/L. She was suspected to have diabetic ketoacidosis (DKA), for which she was treated as per DKA management protocol. Admission blood revealed low hemoglobin with low mean corpuscular volume and raised inflammatory markers (Table 1).

**Table 1:** Laboratory values of the patient during admission

Blood test	First admission	Second admission	Third admission	Normal range
Hemoglobin	90 g/L	105	103	135-180 g/L
White cell count	15.6 x 10⁹/L	6.6 x 10⁹/L	4.9 x 10⁹/L	4.0-11.0 * 10^9^/L
Neutrophil count	13.18 x 10⁹/L	3.85 x 10⁹/L	2.34 x 10⁹/L	2.0-7.0 * 10^9^/L
Platelet count	454 x 10⁹/L	443 x 10⁹/L	393 x 10⁹/L	150-400 * 109/L
Mean corpuscular volume	77.3 fL	81.3	81.4	82-100 fl
C-reactive protein	156 mg/L	10	13	0-5 mg/L
Urea	3.8 mmol/L	8	5.4	2.0-7 mmol/L
Creatinine	43 µmol/L	78	54	55-120 umol/L
HbA1C	135	-	-	-

**Figure 1:**
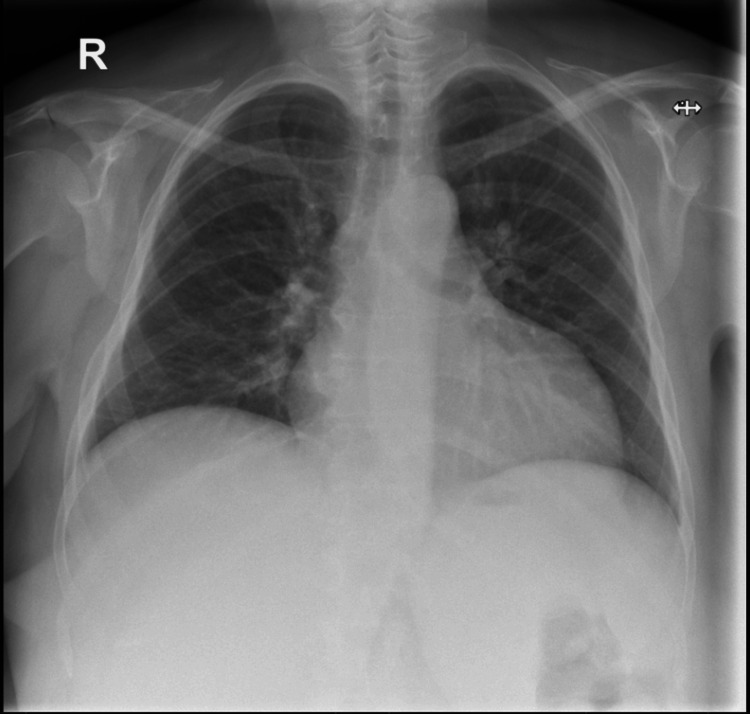
Chest X-ray done on day 1 shows a clear lung field and cardiothoracic ratio of 15/28 cm

Urine dipstick testing showed glucose 2+, ketones 4+, blood 3+, protein 2+, leukocytes positive, and nitrites negative. A chest X-ray was done on arrival to look for the source of infection, which showed a clear lung field and cardiothoracic ratio of 15/28 cm (Figure 1).

**Figure 2:**
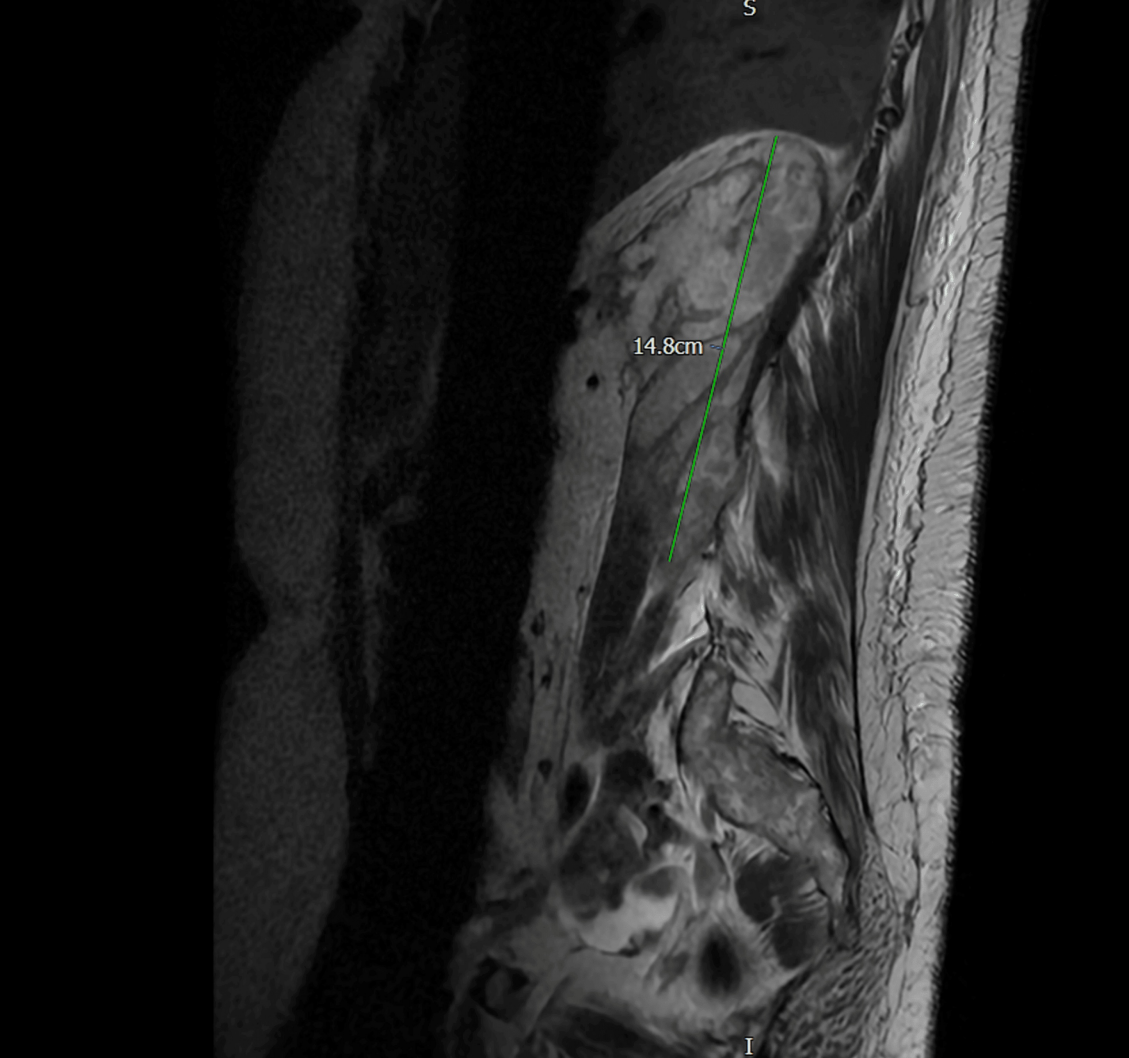
MRI whole spine done on day 2 shows large retroperitoneal collection in the posterior perinephric space and right iliopsoas muscle MRI: magnetic resonance imaging

**Figure 3:**
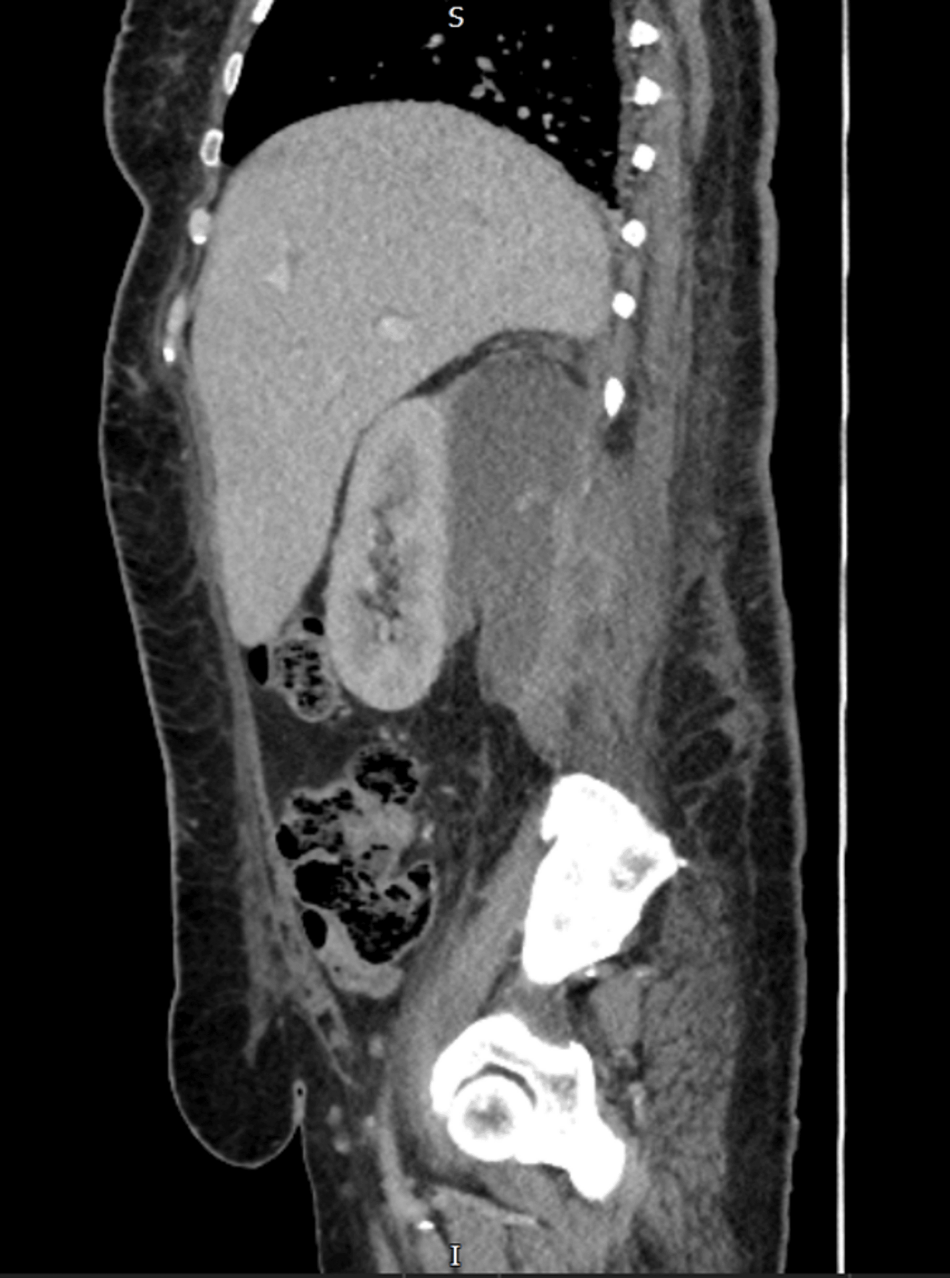
CT abdomen pelvis with contrast sagittal view done on day 3 shows right posterior perinephric collection measuring 7.7 cm x 5.3 cm, extending along the entire length of the kidney, and involving both the right psoas and right erector spinae muscles CT: computed tomography

The patient was admitted to the acute medical unit with a preliminary diagnosis of DKA due to infection and a suspected spinal pathology, likely discitis. The following day, an MRI of the whole spine was performed to investigate the cause of her symptoms and locate the source of the infection. This revealed a large retroperitoneal collection in the posterior perinephric space and right iliopsoas muscle, which was partially imaged on the MRI, measuring at least 15 cm in the craniocaudal dimension. This was likely of urological origin with no spinal abnormalities (Figure 2). Intravenous gentamicin was initiated. A subsequent contrast-enhanced CT scan of the abdomen and pelvis confirmed a right posterior perinephric collection measuring 7.7 cm x 5.3 cm, extending along the entire length of the kidney, and involving both the right psoas and right erector spinae muscles (Figure 3).

**Figure 4:**
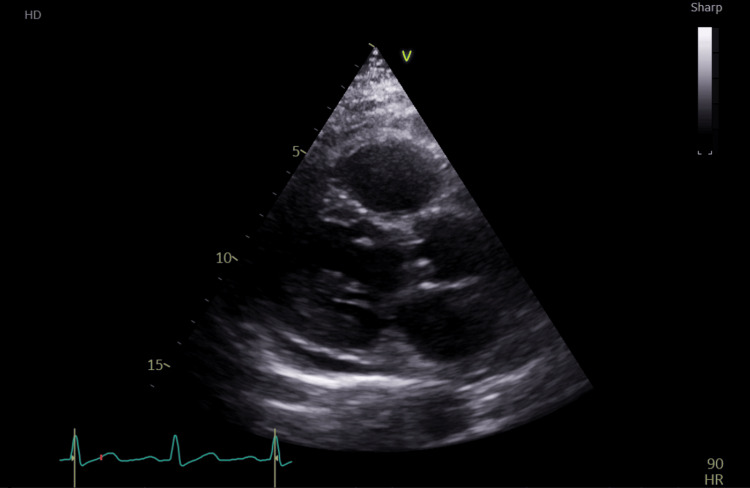
Echocardiography on day 5 of admission shows no evidence of infective endocarditis or vegetations. The LV is of normal size with normal systolic function, with an estimated LVEF of 55%. A small to moderate circumferential pericardial effusion is observed LV: left ventricle, LVEF: left ventricular ejection fraction

An ultrasound-guided drainage procedure was performed on day 3 of admission, during which 200 ml of frank pus was aspirated and flushed with 20 ml of normal saline. An 8-Fr pigtail catheter was then placed for continuous drainage, with instructions for regular flushing with normal saline and removal once the drainage stops. Culture from the drained fluid was sent for further analysis, which grew Klebsiella pneumoniae. The patient’s DKA was precipitated due to underlying infection and suboptimal glycaemic control. A focused echocardiogram was conducted to rule out infective endocarditis as a source of infection and cause for recurrent abscess, which showed a small pericardial effusion but no signs of endocarditis or vegetations (Figure 4).

**Figure 5:**
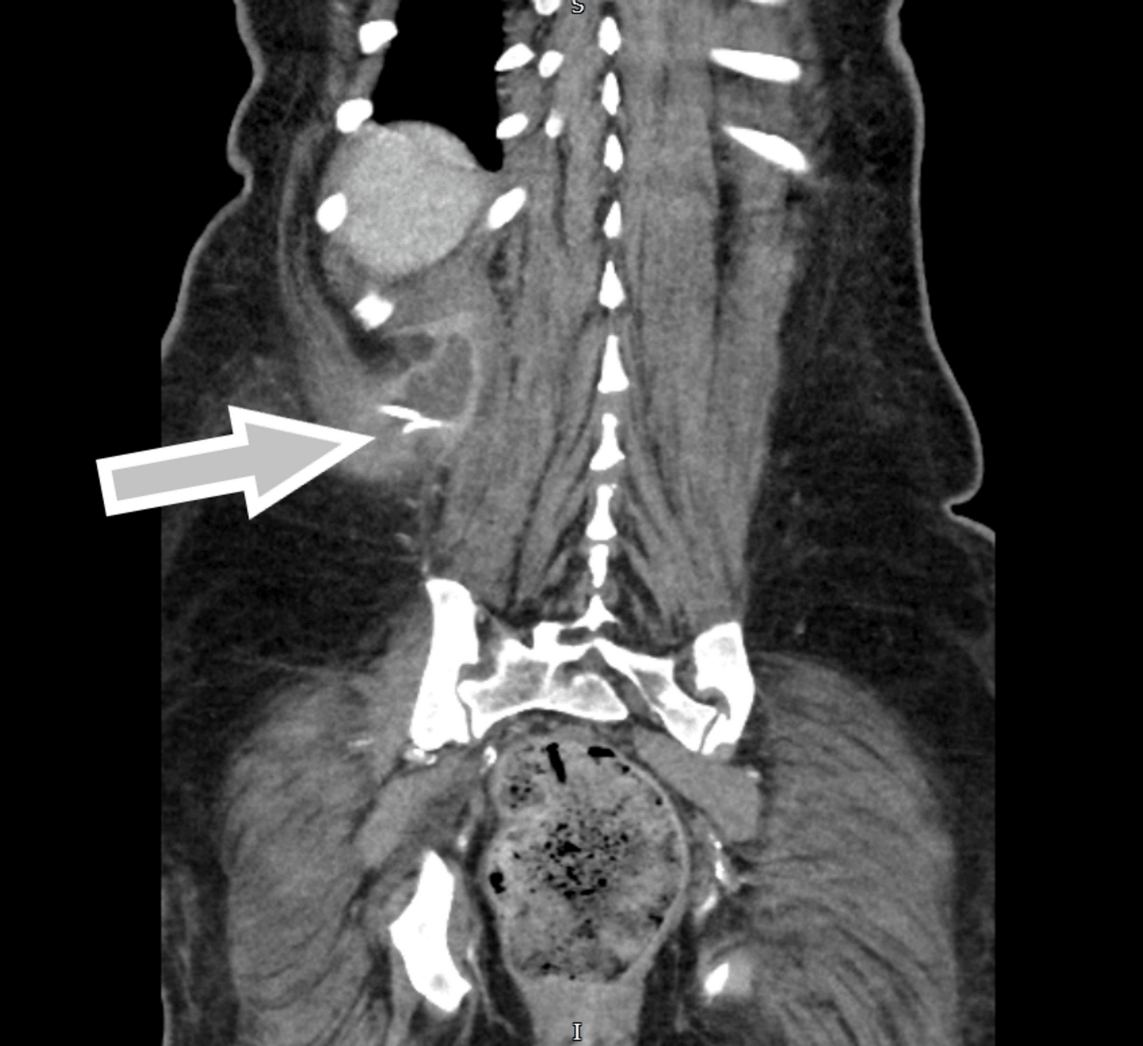
CT abdomen pelvis with contrast following drain insertion shows pigtail catheter within the right perinephric collection, extending into the paravertebral muscles CT: computed tomography

**Figure 6:**
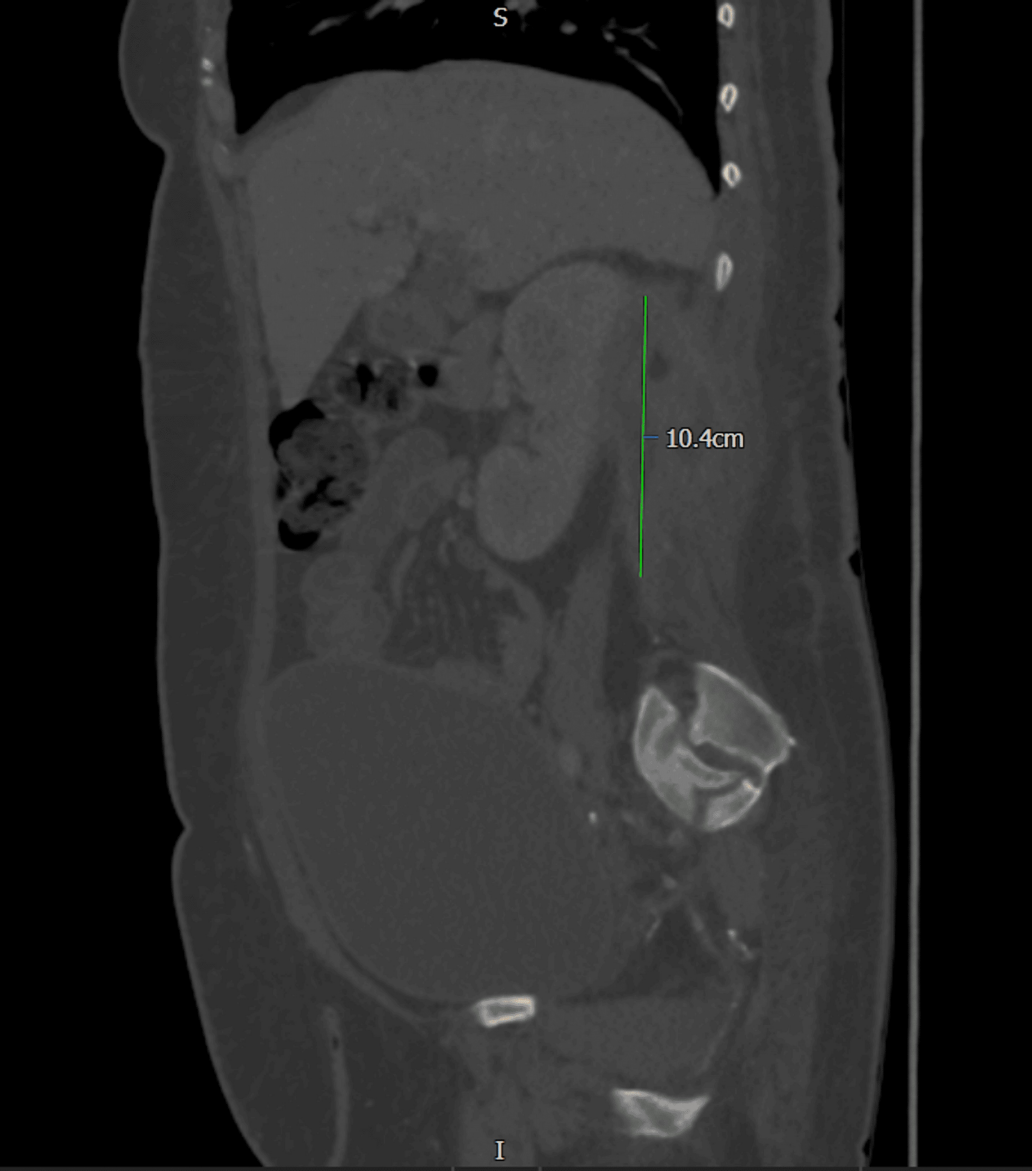
CT abdomen pelvis with contrast done on week 3 of admission shows Rt perinephric collection to be collapsed than before. It has an irregular appearance with multiple ramifications in the soft tissue. The abscess is 3.6 cm x 2 cm in dimension; however, there are soft tissue inflammatory changes surrounding the abscess measuring 10.4 cm x 5.2 cm. This makes the exact measurement of the abscess difficult CT: computed tomography

A follow-up CT scan was performed during the third week of admission to reassess the size of the collection. The scan showed a pigtail catheter within the right perinephric collection, extending into the paravertebral muscles (Figure 5). The collection appeared more collapsed than before, with an irregular shape and multiple branches in the soft tissue. Although exact measurements were difficult, the maximum anteroposterior dimension was approximately 5.2 cm, and the craniocaudal extent was around 10.4 cm (Figure 6).

Later, she developed scalp rashes, and her history of recurrent abscesses raised concerns about a potential underlying immunodeficiency and infection. A referral was sent to infectious diseases, and they advised checking for viral markers, including an assessment of immunoglobulin levels, which revealed an IgM deficiency; viral markers were negative. The scalp rash was treated conservatively with topical permethrin, which resolved completely. An appropriate hematology outpatient referral was sent in view of IgM deficiency and the clinical presentation to further investigate lymphoproliferative disorders.

Ultrasound of the urinary tract was requested due to minimal urine output, which ruled out any calculi/hydronephrosis, and a distended urinary bladder was noted. The patient was on oxybutynin (an antimuscarinic agent) previously for an overactive bladder, which was suspended due to evidence of urinary retention, as it was suspected that the medication might have contributed to this episode. Urinary retention is a known side effect of oxybutynin. The patient subsequently also developed acute kidney injury secondary to urinary retention, which was believed to be the result of autonomic dysfunction associated with poorly controlled diabetes mellitus. She was initially managed with urinary catheterization, followed by a successful trial without a catheter in the community. Additionally, she received education on proper insulin administration to enhance compliance and reduce the risk of future complications. The patient responded well to abscess drainage and concurrent antibiotic therapy based on sensitivity results and was discharged from the hospital with a plan for interventional radiology clinical nurse specialist follow-up within seven days for drain removal and a repeat CT abdomen pelvis in four weeks' time.

The patient presented to the emergency department a week later with complaints of leakage from the drain site. She was reviewed by the urology team, who recommended regular dressing of the site with antibiotic coverage and scheduled a follow-up in the outpatient clinic within two weeks. At this point, she was also awaiting drain removal.

**Figure 7:**
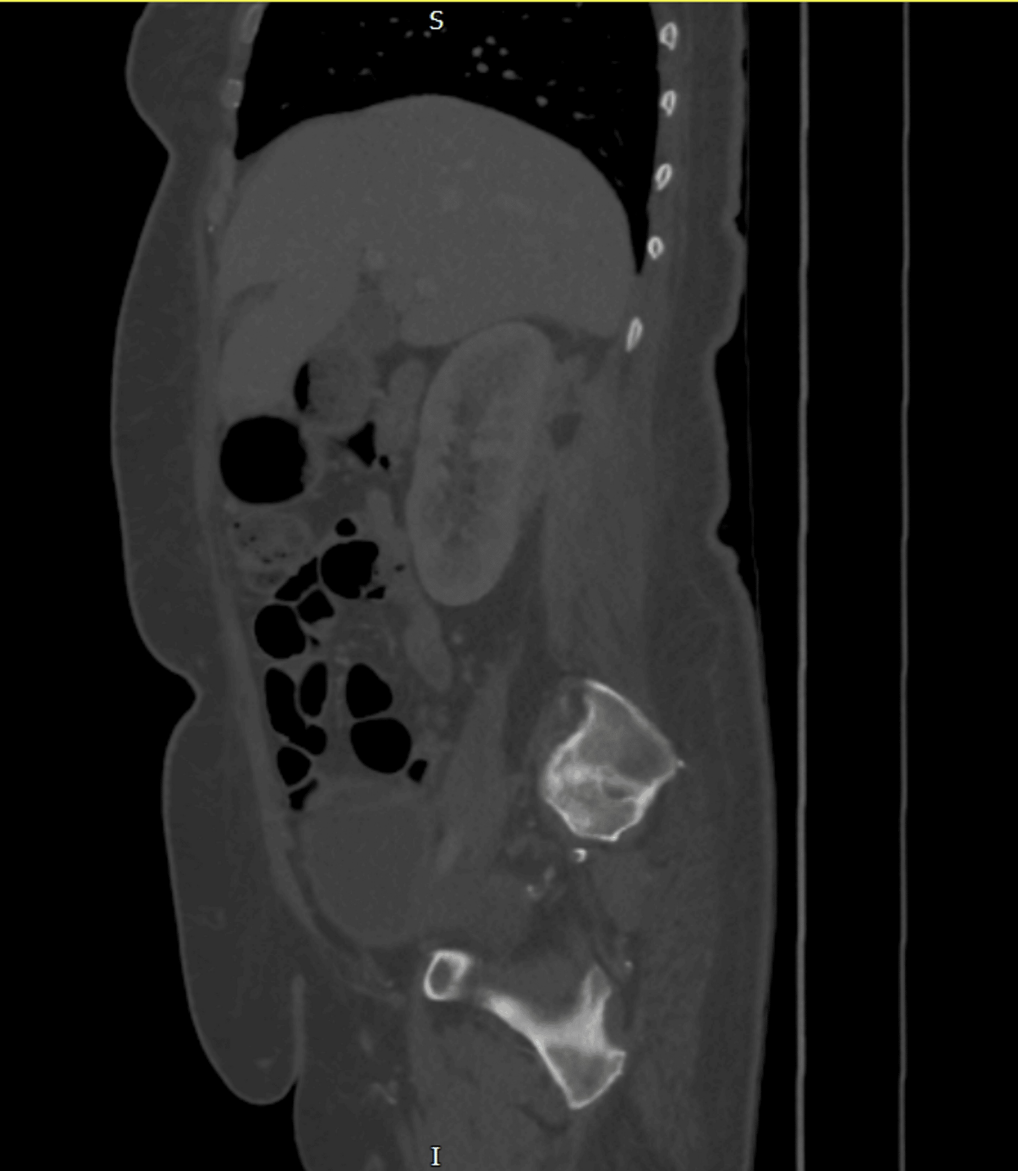
CT abdomen pelvis with contrast shows interval improvement of right perinephric collection. The collection, as before, extends into the paravertebral space but is significantly reduced. Pigtail drain is no longer in situ. The size of the abscess cannot be ascertained as it has collapsed significantly CT: computed tomography

She returned to the emergency department after two weeks with complaints of pain at the drain site accompanied by frank pus, which started immediately after she accidentally pulled out the pigtail drain. Due to pain and frank discharge from the drain site, the patient had to be re-admitted. A CT scan of the abdomen and pelvis with contrast was performed to assess the positioning of the drain and to re-assess the size of the perinephric collection (Figure 7).

The patient was managed conservatively with IV antibiotics, and a follow-up CT scan was arranged in four weeks' time to check for resolution along with an outpatient appointment in eight weeks' time. The patient’s drain cytology was also evaluated for malignancy, which turned out negative.

The patient was later seen in the clinic for follow-up, where she reported occasional right flank pain. Her persistent storage and voiding urinary symptoms were assessed, and she was advised to restart oxybutynin and to consider intermittent catheterization for symptomatic management. A follow-up ultrasound of the urinary tract has been scheduled to monitor for any re-accumulation of the abscess. She is also scheduled for a further outpatient review in two months and awaiting a hematology outpatient review to investigate any evidence of lymphoproliferative disorders.

## Discussion

The psoas muscle originates from the lateral processes of T12 to L5 and inserts at the lesser trochanter of the femur along with the iliacus muscle via the iliopsoas tendon. The muscle is a major hip flexor. Psoas muscle is highly vascular, supplied by the lumbar, iliolumbar, obturator, external iliac, and common femoral arteries, which makes them active targets for hematogenous infections leading to primary abscesses. Infection can also spread to the psoas muscle from surrounding structures, including vertebral bodies, the abdominal aorta, and the GI tract, including the sigmoid colon, appendix, hip joint, and iliac lymph nodes, leading to secondary abscess [1]. Secondary abscesses are more common than primary abscesses [2].

The causative organisms are usually *Staphylococcus aureus* and less commonly *Escherichia coli*, *Klebsiella pneumoniae*, and *Streptococcus*. *Mycobacterium tuberculosis* is also a major cause, particularly in developing countries [3].

Primary abscesses are more common in developing countries, particularly in populations at risk, including children, immunocompromised individuals, diabetics, drug users, and patients with chronic renal failure. In our case, the patient had IgM deficiency and longstanding uncontrolled diabetes, which caused her to have recurrent multiple abscesses involving various parts of her body. In such cases, infective endocarditis is an important complication due to hematogenous seeding.

Patients with diabetes mellitus have an increased susceptibility to infections due to multiple factors, including impaired neutrophil migration, reduced phagocytic activity, and compromised humoral immunity [4]. Additionally, diabetes can lead to enhanced adherence of pathogens to host cells, along with underlying complications such as neuropathy and microangiopathy, which further increase vulnerability to various complications, including chronic infections, delayed wound healing, and an increased risk of cardiovascular events. DKA is an acute, major, life-threatening complication of diabetes characterized by hyperglycemia, ketoacidosis, and ketonuria. Poor glycemic control exacerbates these issues, creating an environment that facilitates infection [5].

Patients with long-standing diabetes mellitus may develop autonomic neuropathy, leading to neurogenic bladder, characterized by reduced bladder sensation and impaired emptying, which can further lead to an increase in post-void residual volume and recurrent urinary tract infections [6,7].

The clinical presentation of IPA is usually sub-acute and non-specific [8]. Classically, a triad of fever, back pain, and limp is described; however, due to its proximity to the lumbar plexus, patients can also present with atypical neurological features, including signs and symptoms of LMN lesions, as in this case. The lumbar plexus consists of nerves responsible for the sensory and motor functions of the lower limbs. An abscess within the psoas muscle can cause nerve compression, leading to decreased motor power, reduced reflexes, and even sensory changes, which are characteristics of LMN lesions [9]. Untreated IPA can lead to surgical emergencies like cauda equina syndrome [10]. The diagnosis is challenging and requires early consideration of its possibility and the use of proper radiological imaging techniques.

The management is usually ultrasound-guided percutaneous drainage and antibiotics. Sometimes surgical drainage of the abscess is required if it’s not amenable to percutaneous drainage or the size is bigger. The surgical approach provides access for debriding the infected tissues and so less chance of recurrence but is associated with higher procedure-related complications [11]. In our case, the urology team preferred percutaneous drainage, which was less invasive and a better choice considering the immunocompromised status of the patient.

Bacterial and fungal infections from the urinary tract can spread directly to the psoas sheath. There are few studies that account for bacterial infection after the endourological procedure as a cause of the direct spread of infection to the psoas muscle [12]. Infections from the urinary system can reach the psoas directly, while infections from distant sites spread via the lymphatic or hematogenous system.

## Conclusions

The IPA is a condition that usually affects immunocompromised individuals and presents in a perplexing way. This case emphasizes the need for a thorough diagnostic approach when encountering atypical neurological presentations and early appropriate radiological imaging to confirm the diagnosis. IPA may rarely extend to the epidural space, causing surgical emergency of cauda equina syndrome. It’s also important to investigate immunodeficiency status in the right group of patients, as this can be a major risk factor for hematogenous or lymphatic spread of the infection. Thus, early diagnosis and management are essential for favorable outcomes. The management requires drainage of the abscess and concurrent antibiotic therapy.
